# Supplementary material for: Dietary EPA+DHA Mitigate Hepatic Toxicity and Modify the Oxylipin Profile in an Animal Model of Colorectal Cancer Treated with Chemotherapy
Source: Cancers (Basel). 2022 Nov 21;14(22):5703. doi: 10.3390/cancers14225703 (PMC9688617; doi:10.3390/cancers14225703)
Supplement: Supplementary file 1 [file cancers-14-05703-s001.zip › cancers-1984620-supplementary.pdf]

Supplemental Table S1: Liver oxylipins in reference, tumor, chemotherapy, and fish oil group animals.

Oxylipins (pg/mg)	PUFA	Reference	Tumor	Chemo	Fish Oil	P-value
9-HODE	LA	116±18.3[a]	143±15.1[a]	133±30.4[a]	79.1±18.8[b]	<.001
9-OxoODE		108±14.7	89.5±17.4	88.6±21.8	62.2±21.6	0.016
13-HODE		116±25.7[a]	122±10.4[a]	127±29.4[a]	68.9±15.0[b]	<.001
13-OxoODE		31.1±8.42[a]	27.3±8.51[a]	24.1±7.30[a]	13.8±2.92[b]	0.004
9,10,13-TriHOME		50.0±11.3[a]	50.7±8.42[a]	66.4±29.7[a]	28.4±2.52[b]	0.002
9,12,13-TriHOME		16.2±2.94[a]	16.7±2.71[a]	22.0±10.9[a]	9.56±1.21[b]	0.002
9,10-DiHOME		11.2±4.65	11.9±3.77	14.3±5.75	5.87±1.16	0.015
12,13-DiHOME		7.32±2.02	6.71±0.93	6.25±1.55	4.97±1.82	0.126
9,10-EpOME		2.75±1.45	2.30±0.99	1.66±0.64	1.02±0.54	0.013
13-HOTrE-γ	GLA	5.89±1.82[a]	6.74±1.45[a]	5.47±1.65[a]	1.99±0.41[b]	<.001
PGD_1	DGLA	1.44±0.42[a]	1.31±0.26[a]	1.34±0.39[a]	0.32±0.29[b]	<.001
15k-PGE_1		0.17±0.03[a]	0.17±0.02[a]	0.15±0.01[a]	0.11±0.03[b]	<.001
8-HETrE		2.35±0.53[a]	2.09±0.65[ab]	1.58±0.44[bc]	1.26±0.33[c]	0.006
15-HETrE		9.76±2.43[a]	11.0±1.29[a]	9.17±2.57[a]	6.33±1.23[b]	0.006
PGA_2	AA	0.64±0.26[b]	0.68±0.22[b]	1.56±1.36[a]	0.69±0.38[b]	<.001
PGD_2		43.8±7.52[a]	36.7±8.01[a]	46.9±17.5[a]	13.8±4.59[b]	<.001
15Δ-PGD_2		0.12±0.03	0.14±0.03	0.19±0.11	0.10±0.06	0.112
PGE_2		16.3±4.46[a]	15.1±1.94[a]	17.5±4.16[a]	5.51±1.17[b]	<.001
11β-PGE_2		4.60±1.17[a]	4.10±0.51[a]	4.69±1.07[a]	1.53±0.34[b]	<.001
15k-PGE_2		6.44±2.06[a]	5.71±1.09[a]	5.41±1.18[a]	2.27±0.61[b]	<.001
$PGF_{2\alpha}$		48.0±12.0[a]	45.8±5.34[a]	51.8±10.9[a]	16.8±2.86[b]	0.003
Dhk-$PGF_{2\alpha}$		1.29±0.58[ab]	2.15±0.41[a]	1.50±0.45[ab]	0.27±0.59[b]	<.001
6k-$PGF_{1\alpha}$		4.37±1.60[b]	4.47±0.78[b]	6.32±3.69[a]	1.72±0.44[c]	<.001
15k-$PGF_{2\alpha}$		22.3±6.26[a]	21.8±2.86[a]	21.7±4.74[a]	8.69±2.49[a]	<.001
PGJ_2		0.40±0.13	0.42±0.13	0.83±0.69	0.40±0.20	0.381
15Δ-PGJ_2		1.67±1.14	1.66±0.93	5.20±4.00	1.31±1.08	0.095
TXB_2		19.7±3.30[ab]	15.4±2.38[b]	24.8±8.50[a]	5.87±2.22[c]	<.001
12-HHTrE		289±49.4[a]	298±56.0[a]	316±57.4[a]	102±25.7[b]	<.001
5-HETE		21.9±1.73[a]	17.8±1.93[b]	16.5±1.57[b]	10.3±1.28[c]	<.001
5-OxoETE		8.46±1.49[a]	5.98±1.46[b]	6.40±1.68[b]	3.59±0.75[c]	<.001
8-HETE		27.0±5.00[a]	24.2±7.72[ab]	18.1±6.24[bc]	11.9±2.53[c]	0.001
9-HETE		29.0±6.11[a]	22.7±5.04[b]	21.2±4.46[b]	14.0±3.57[c]	<.001
11-HETE		29.1±8.32	29.3±5.36	25.2±8.49	16.7±3.95	0.018
12-HETE		48.4±11.5[a]	39.7±7.90[a]	27.8±8.52[ab]	17.0±6.93[c]	<.001
12-OxoETE		46.1±14.3[a]	32.7±8.28[b]	31.6±9.78[b]	13.3±2.26[c]	<.001
15-HETE		43.9±6.83[a]	47.7±8.13[a]	45.6±17.1[a]	26.7±5.83[b]	<.001
15-OxoETE		5.24±1.33[a]	3.68±0.96[b]	3.49±1.16[b]	2.53±0.73[b]	0.004
5,15-DiHETE		0.82±0.23	0.94±0.39	0.77±0.15	1.94±1.50	0.663

8,15-DiHETE		7.96±1.28	8.62±2.64	8.09±2.00	7.65±2.54	0.895
5,6-DiHETrE		1.71±0.52[a]	1.39±0.22[ab]	1.07±0.13[bc]	0.96±0.31[c]	0.002
8,9-DiHETrE		4.65±1.72	4.47±0.91	5.41±1.49	3.02±0.77	0.039
11,12-DiHETrE		5.42±1.93	5.03±1.15	6.56±2.81	2.65±0.79	0.017
14,15-DiHETrE		5.31±1.40	4.72±0.61	5.37±1.86	3.25±0.98	0.048
5,6-EpETrE		4.66±1.01[a]	4.05±1.28[a]	3.84±0.93[a]	1.85±0.53[b]	<.001
11,12-EpETrE		2.02±0.84[a]	1.63±0.61[ab]	1.14±0.50[bc]	0.55±0.19[c]	0.002
14,15-EpETrE		0.58±0.34	0.40±0.11	0.31±0.17	0.24±0.13	0.030
16-HETE		15.3±3.08	22.4±6.19	14.9±7.94	14.9±9.10	0.222
17-HETE		0.82±0.27	0.87±0.34	0.73±0.56	0.74±0.48	0.938
18-HETE		1.67±0.45	1.18±0.42	1.16±0.38	1.23±0.55	0.207
20-HETE		11.0±1.41	9.55±1.41	8.86±1.51	7.47±3.46	0.064
8-Iso-$PGF_{2\alpha}$III*		1.35±0.21[a]	1.37±0.16[a]	1.39±0.42[a]	0.74±0.29[b]	0.003
9-HOTrE	ALA	3.54±0.59[a]	4.53±0.66[a]	4.12±1.26[a]	2.15±0.40[b]	<.001
9-OxoOTrE		2.69±1.64	1.57±0.50	1.68±0.59	2.15±0.64	0.295
13-HOTrE		4.02±0.74[b]	5.33±1.02[a]	4.54±0.62[ab]	2.87±0.66[c]	<.001
PGE_3	EPA	1.49±0.43[b]	1.45±0.53[b]	1.48±0.46[b]	6.38±3.19[a]	0.005
$PGF_{3\alpha}$		2.24±0.67[b]	1.61±0.33[b]	1.48±0.27[b]	7.26±3.25[a]	0.001
TXB_3		0.19±0.13[bc]	0.01±0.07[d]	0.12±0.08[bcd]	1.23±0.42[a]	<0.001
8-HEPE		1.95±0.92[b]	2.10±0.58[b]	1.98±0.50[b]	15.6±2.90[a]	0.004
11-HEPE		5.62±2.18[b]	5.15±1.19[b]	2.78±1.00[c]	30.0±12.7[a]	<0.001
12-HEPE		6.91±1.41[b]	5.23±1.15[b]	2.35±0.67[c]	30.7±9.81[a]	<.001
15-HEPE		5.53±2.26[b]	5.21±1.27[b]	3.70±1.43[b]	51.2±22.9[a]	0.002
14,15-DiHETE		21.9±8.15[b]	20.4±2.52[b]	17.9±6.36[b]	241±51.5[a]	0.005
17,18-DiHETE		16.7±5.88[b]	15.6±2.34[b]	15.2±2.59[b]	205±61.4[a]	0.004
18-HEPE		4.55±1.43[b]	4.12±1.48[b]	3.29±0.91[b]	49.5±12.6[a]	0.002
4-HDoHE	DHA	14.8±2.11[b]	14.4±2.59[b]	11.5±1.49[b]	18.4±4.46[a]	0.005
7-HDoHE		6.37±0.82[b]	6.89±2.57[b]	5.66±1.58[b]	12.7±1.77[a]	<.001
8-HDoHE		16.7±3.77	13.5±3.28	11.8±3.75	19.9±6.32	0.028
10-HDoHE		4.68±0.92[bc]	5.11±1.06[b]	3.71±0.58[c]	6.68±2.72[a]	<.001
11-HDoHE		7.43±1.18[b]	7.67±1.53[b]	5.98±0.99[b]	9.94±2.86[a]	0.010
13-HDoHE		12.3±4.91[b]	11.1±1.23[b]	9.45±4.04[c]	16.8±4.11[a]	<.001
14-HDoHE		9.55±1.63[b]	10.5±2.78[ab]	7.46±2.22[c]	14.5±4.52[a]	0.006
16-HDoHE		7.94±1.11[b]	7.80±1.34[b]	5.98±0.91[c]	11.1±2.34[a]	<.001
17-HDoHE		24.1±5.93[ab]	28.3±7.72[a]	20.4±4.60[bc]	35.9±11.4[a]	0.003
16,17-DiHDoPE		0.87±0.19[b]	0.83±0.11[b]	1.05±0.12[b]	1.34±0.35[a]	0.003
19,20-DiHDoPE		2.30±0.75	2.43±0.44	2.42±0.92	3.22±1.05	0.254
20-HDoHE		11.8±1.99[b]	11.1±1.81[b]	9.86±2.19[b]	16.7±3.64[a]	<.001

Values are expressed as mean ng/g tissue ± SD. Differing lower case superscript letters indicate significant simple effect differences between values ($p<0.008$). Reference, healthy rats did not undergo tumor implantation nor received chemotherapy, consumed control diet only; Tumor, tumor bearing

animals did not receive chemotherapy, consumed control diet only; Chemo, tumor bearing animals received chemotherapy, consumed control diet only; Fish Oil, tumor bearing animals received chemotherapy, consumed fish oil diet.

Abbreviations: AA, Arachidonic acid; ALA, Alpha-linolenic acid; DγLA, Dihomo-gamma-linolenic acid; DHA, Docosahexaenoic acid; DiHDoHE, Dihydroxy-docosahexaenoic acid; DiHDPE, Dihydroxy-docosapentaenoic acid; DiHETrE, Dihydroxy-eicosatrienoic acid; DiHOME, Dihydroxy-octadecenoic acid; EPA, Eicosapentaenoic acid; HDoHE, Hydroxy-docosahexaenoic acid; HEPE, Hydroxy-eicosapentaenoic acid; HETE, Hydroxy-eicosatetraenoic acid; HETrE, Hydroxy-eicosatrienoic acid; HODE, Hydroxy-octadecadienoic acid; HOTrE,Hydroxy-octadecatrienoic acid; k, keto; LA, Linoleic acid; oxo-ETE, oxo-Eicosatetraenoic acid; oxo-ODE, oxo-Octadecadienoic acid; oxo-OTrE, oxo-Octadecatrienoic acid; PG, Prostaglandin; PUFA, polyunsaturated fatty acid; TriHOME, Trihydroxy-octadecenoic acid.

Supplemental Table S2: Liver cytokines.

	Reference	Tumor	Chemo	Chemo+Fish Oil	P-value
Eotaxin	20.63±1.54[ab]	21.05±2.66[ab]	22.48±0.77[a]	18.81±2.41[b]	0.021
IFN-γ	370.1±21.2	376.7±23.3	374.8±73.1	388.9±40.0	0.872
IL-1α	154.8±15.0	167.4±13.6	186.4±48.7	181.1±16.2	0.122
IL-1β	570.3±47.9	574.4±40.2	623.1±48.5	564.1±48.3	0.108
IL-6	2956±507[b]	2541±376[b]	3766±793[a]	2857±688[b]	0.009
IL-10	296.6±35.9[ab]	272.5±28.2[b]	323.1±13.5[a]	293.0±31.8[ab]	0.038
IL-17A	12.65±3.71	12.94±1.72	16.3±4.84	13.80±3.84	0.273
IL-18	609.2±79.9[ab]	662.8±49.9[a]	520.8±85.9[bc]	494.0±33.8[c]	<.001
TNF-α	11.53±2.11[b]	8.560±1.06[c]	16.49±2.54[a]	15.26±2.46[a]	<.002

Values are expressed as mean pg/mL tissue ± SD. Differing lower case superscript letters indicate significant simple effect differences between values (p<0.05). Reference, healthy rats did not undergo tumor implantation nor received chemotherapy, consumed control diet only; Tumor, tumor bearing animals did not receive chemotherapy, consumed control diet only; Chemo, tumor bearing animals received chemotherapy, consumed control diet only; Fish Oil, tumor bearing animals received chemotherapy, consumed fish oil diet.

MCP-1 was below the detection limit of the assay for all groups.

Abbreviation: IFN-γ, interferon gamma; IL, interleukin; MCP-1, monocyte chemoattractant protein 1; TNF-α, tumor necrosis factor alpha.
